# Non-invasive evaluation of pulmonary arterial blood flow and wall shear stress in pulmonary arterial hypertension with 3D phase contrast magnetic resonance imaging

**DOI:** 10.1186/s40064-016-2755-7

**Published:** 2016-07-13

**Authors:** Keiichi Odagiri, Naoki Inui, Akio Hakamata, Yusuke Inoue, Takafumi Suda, Yasuo Takehara, Harumi Sakahara, Masataka Sugiyama, Marcus T. Alley, Tetsuya Wakayama, Hiroshi Watanabe

**Affiliations:** Department of Clinical Pharmacology and Therapeutics, Hamamatsu University School of Medicine, 1-20-1 Handayama, Higashi-ku, Hamamatsu, 431-3192 Japan; Department of Internal Medicine II, Hamamatsu University School of Medicine, Hamamatsu, Japan; Department of Radiology, Hamamatsu University School of Medicine, Hamamatsu, Japan; Department of Radiology, Stanford University, Palo Alto, CA USA; GE Healthcare Japan, Hino, Japan

**Keywords:** 4D-flow, Pulmonary artery, Diagnosis, Magnetic resonance imaging, Right ventricle

## Abstract

**Background:**

Recently, time-resolved 3D phase contrast magnetic resonance imaging (4D-flow) allows flow dynamics in patients with pulmonary arterial hypertension to be measured. Abnormal flow dynamics, such as vortex blood flow pattern in the pulmonary artery (PA), may reflect progression of pulmonary arterial hypertension (PAH). Some reports suggested that abnormal blood flow parameters including wall shear stress (WSS) could be markers of PAH. However, it was not fully assessed clinical usefulness of these variables. We aimed to assess whether these flow dynamic parameters, such as vortex formation time (VFT) and WSS, were associated with right ventricular (RV) function.

**Results:**

Fifteen subjects, nine with PAH and six healthy volunteers, underwent 4D-flow. Differences of Blood flow patterns, blood flow velocities and WSS between PAH patients and healthy volunteers were evaluated. We also assessed the association between VFT, WSS and RV function in PAH patients. Both vortex blood flow patterns and early systolic retrograde flow in the main PA were observed in all patients with PAH. The PA flow velocities and WSS in patients with PAH were lower than those in healthy volunteers, but that blood flow volumes in the MPA, RPA and LPA and SV in the MPA were broadly comparable between the groups. The mean VFT was 35.0 ± 16.6 % of the cardiac cycle. The VFT significantly correlated with RV ejection fraction, RV end systolic volume, and RV end systolic volume index (RVEF = 75.1 + (−85.7)·VFT, p = 0.003, RVESV = 12.4 + 181.8·VFT, p = 0.037 and RVESVI = 10.6 + 114.8·VFT, p = 0.038, respectively) in PAH patients, whereas WSS did not correlate with RV function.

**Conclusions:**

We confirmed that abnormal blood flow dynamics, including the vortex formation and the early onset of retrograde flow, low WSS in the PA were characteristics of PAH. The VFT may be associated with right ventricular dysfunction, whereas WSS was not. Our results suggest that 4D-flow is an effective means of detecting right heart failure as well as diagnosing PAH.

*Clinical trial registration* URL: https://upload.umin.ac.jp/cgi-open-bin/ctr/ctr.cgi. Unique identifier: UMIN000011128

**Electronic supplementary material:**

The online version of this article (doi:10.1186/s40064-016-2755-7) contains supplementary material, which is available to authorized users.

## Background

Pulmonary arterial hypertension (PAH) is a progressive disease with a poor prognosis characterized by increasing pulmonary vascular resistance and pulmonary arterial pressure (PAP) (D’Alonzo et al. 1991; Runo and Loyd 2003). The raised mean pulmonary arterial pressure provokes right heart failure which is the primary cause of death in patients with PAH (D’Alonzo et al. 1991). In recent years, several drugs, such as phosphodiesterase-5 (PDE-5) inhibitors, endothelin receptor antagonists (ERA), and prostanoids approved for the treatment of PAH have been shown to improve the symptoms, exercise tolerance and mortality of patients with PAH (Rubin et al. 2002; Galie et al. 2005; Rubin et al. 2011; Pulido et al. 2013). As prompt treatment with these drugs is associated with better treatment outcomes, it is essential that the diagnosis of PAH is not delayed (McGoon et al. 2004).

Nonetheless, the early detection and diagnosis of PAH is challenging, as PAH is likely to be asymptomatic until pulmonary vascular lesions have progressed. Echocardiography (UCG) is usually performed when PAH is suspected; however, Doppler-derived pressure estimation is often inaccurate (Fisher et al. 2009). Consequently, estimation of PAP by Doppler UCG is not recommended as a screening technique to detect mild, asymptomatic PAH (Galie et al. 2009). Cardiac magnetic resonance imaging (CMR) is a non-invasive means of evaluating right ventricular (RV) function and the characteristics of the pulmonary vascular bed, and can also detect myocardial fibrosis (McLaughlin et al. 2009; Bradlow et al. 2012). Furthermore, in recent years, time-resolved 3-dimensional (3D) phase-contrast magnetic resonance imaging (4D-flow MRI) allows pulmonary intravascular blood flow to be visualized, and blood flow velocity, volume and wall shear stress (WSS) to be measured (Barker et al. 2014; Helderman et al. 2011; Ota et al. 2015; Reiter et al. 2008; Tang et al. 2012; Truong et al. 2013). This new technology revealed that the abnormal blood flow dynamics, including the vortex formation and the early onset of retrograde flow were characteristics of PAH, and the vortex formation time (VFT) was correlated with PAP (Reiter et al. 2008, 2015). Although investigators revealed that low values of WSS in pulmonary artery in PAH patients were lower than those in healthy volunteers, it was not established clinical usefulness of these variables (Truong et al. 2013; Barker et al. 2014). In this study, we aimed to assessed the relationships between flow dynamic and hemodynamic parameters, including VFT and WSS obtained by 4D-flow and RV functional parameters measured by CMR.

## Methods

### Ethics statement

Our study protocol complied with the Declaration of Helsinki and was approved by the institutional research review board of Hamamatsu University School of Medicine, Hamamatsu, Japan. Written informed consent was provided by all subjects. The study was registered at the UMIN Clinical Trials Registry (UMIN 000011128).

### Study design and subjects

Nine patients with PAH (defined as mean PAP > 25 mmHg measured by right heart catheter (RHC) at the time of their diagnosis) in World Health Organization (WHO) functional class II–III and six healthy volunteers (without known significant health problems, including heart or lung diseases, or symptoms) were enrolled in this study. All underwent a gadolinium-enhanced magnetic resonance angiography (MRA) and 4D-flow at Hamamatsu University Hospital between December 2012 and September 2014. Exclusion criteria were atrial fibrillation and atrial flutter.

### MR imaging

All MR imaging was performed using a Discovery 750, 3T machine (GE Healthcare, Waukesha, WI, USA) together with a 32-channel phased array torso coil. The gradient performance had a maximum gradient strength of 50 mT/m and a maximum slew rate of 200 mT/m/ms.

### Time-resolved contrast-enhanced 3D MRA and 4D-flow

Contrast-enhanced breath-hold three-dimensional MRA (Gd 3D MRA) was repeated before and after bolus injection of gadolinium chelate with standard dose of 0.1 mmol/kg at an injection rate of 2–3 ml/s using an auto-injector. The contrast agent was used to increase the definition of the arterial wall boundary for post-processing and also to increase the signal-to-noise ratio in the 4D-flow measurement. The data acquisitions for contrast enhanced MR angiogram was performed during breath-holding at neutral position, which was not in the inhaled position or exhaled position.

Electrocardiogram-gated, respiratory-compensated coronal 3D fast spoiled gradient-recalled echo in the steady state (FSPGR)-based 4D-flow was conducted covering the right atrium, right ventricle and proximal portion of the PA with the parameters described in the data supplement (Supplementary method in Additional file 1).

4D-flow and MRA data sets were transferred to a personal compute in DICOM format, and were post-processed with flow analysis software (Flova; R Tech, Hamamatsu, Japan). The application consisted of two processes for data extraction and analysis. The application consisted of two processes of extraction and analysis. First, time-resolved images of 3D velocity vector fields were generated to overview the blood flow within the abdominal aorta. Then, 3D streamlines, wall shear stress (WSS) maps were generated using 4D data sets. Flow vector data acquired with 4D-flow was segmented by using the vessel boundary information determined by contrast enhanced MRA. Time-resolved images of 3D velocity vector fields were generated to obtain an overview of the blood flow within the PA. Second, color-coded 3D streamlines and WSS maps were generated using the 4D data sets.

### Generation of 3D streamlines and calculation of WSS

**Fig. 1:**
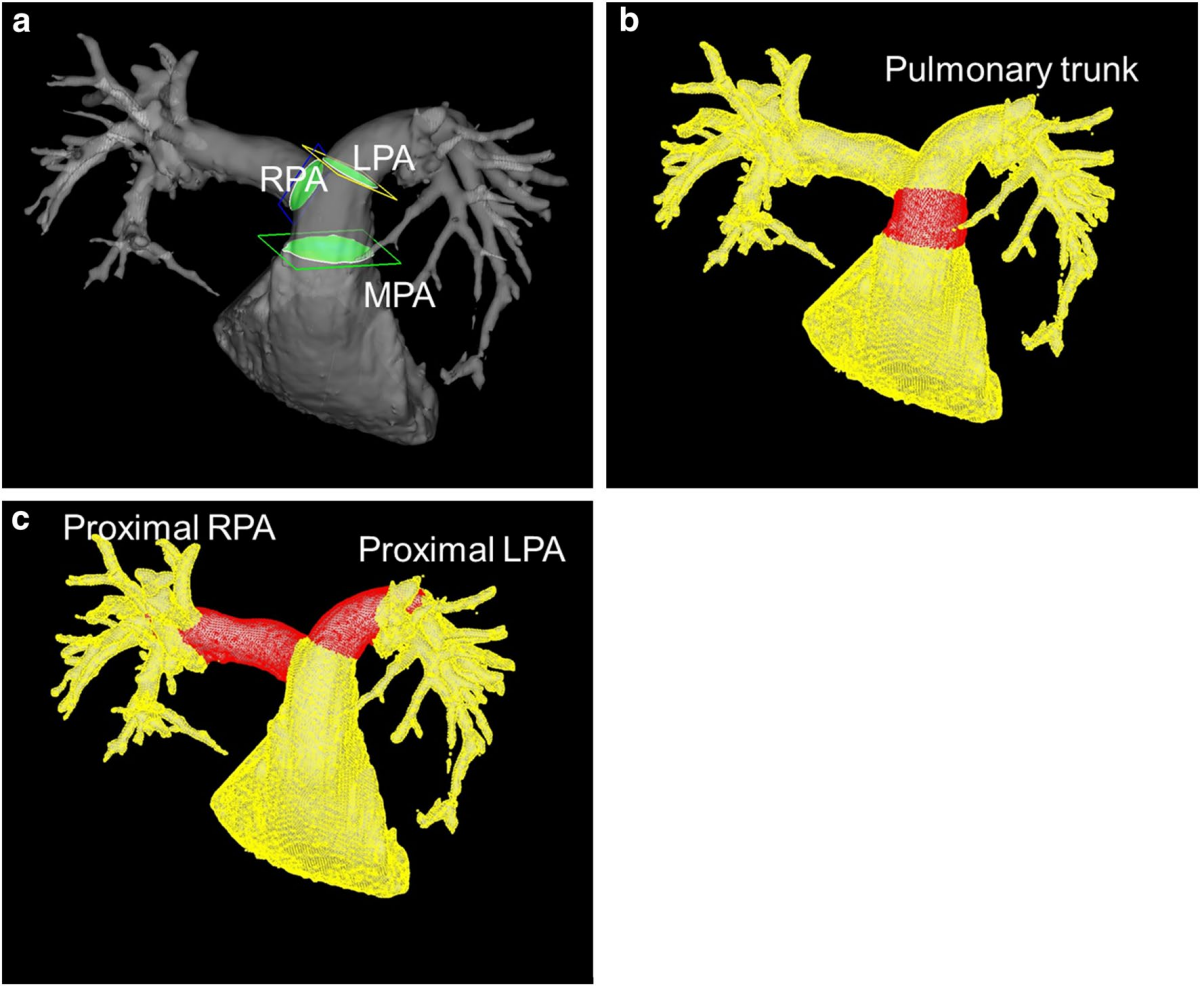
Cross-sectional planes traversing the main, right and left pulmonary artery and the locations of the pulmonary trunk, proximal RPA and proximal LPA. **a** Cross-sectional planes traversing the main, right and left pulmonary artery. All cross-sectional planes were placed perpendicular to the longitudinal axis of the pulmonary artery. Each cut plane was placed immediately downstream of the pulmonary valve cusps or the bifurcation of the main pulmonary artery. **b**, **c** The locations of the pulmonary trunk (**b**), proximal RPA and proximal LPA (**c**), in which wall shear stress values were calculated. *MPA* main pulmonary artery, *RPA* right pulmonary artery, *LPA* left pulmonary artery, *RPA* right pulmonary artery, *LPA* left pulmonary artery

We chose several cross-sectional planes traversing the main PA (MPA), right PA (RPA) and left PA (LPA). All planes were placed perpendicular to the longitudinal axis of the PA. Figure 1a indicates the locations of the MPA, RPA or LPA cross-sectional planes, which were placed immediate downstream of pulmonary valve cusps or RPA/LPA bifurcation, respectively. We then generated 3D streamlines to visualize blood flow pattern in the MPA using the Runge–Kutta method (Liu et al. 2006).

Wall shear stress is defined as the product of fluid viscosity and shearing velocity of the blood in the vicinity of the vascular wall. The method we used to calculate the shearing velocity vector was similar to that reported by Masaryk et al. (1999) and Cheng et al. (2002), however, our method was 3D instead of 2D. A 3D method to measure WSS has previously been reported (Isoda et al. 2010) on which our application is based. The dynamic viscosity of the blood was assumed to be 0.00384 Pa/s in the application (please refer to data supplement in Additional file 1 for further details on the method used).

### Analyses of right ventricular function

Breath-hold cine magnetic resonance images were obtained in contiguous short-axis planes from the cardiac apex to the base with the patient in a resting state. The 2D fast imaging employing steady state acquisition (FIESTA) cine images were based on the steady state free precession sequence.

### Image analysis and measurements

The diameters and areas of the PA were measured at the cross-sectional cut planes. Blood flow patterns were analyzed visually for the presence of vortex blood flow and early systolic retrograde flow in the MPA. We excluded the valvular vortical and helical flow from the analysis. These blood flow patterns were determined by two observers. The VFT (% of the cardiac cycle) in the MPA was also calculated (number of the cardiac phase with vortex divided by 20) with frame-counting method by single observer. Maximum and mean blood flow volumes, velocities which passed the cross-sectional planes for the MPA, RPA and LPA were measured automatically. Stroke volumes (SV) at the MPA, RPA and LPA were calculated by temporal integration of blood flow volumes. WSS values in the pulmonary trunk, proximal RPA or LPA were also calculated automatically (Fig. 1b, c). Right ventricular end diastolic volume (RVEDV), RV end systolic volume (RVESV) and RV ejection fraction (RVEF) were determined from 2D FIESTA cine images in the short axis view. They were automatically calculated with analysis software (CardiacVX, GE Healthcare, Waukesha, WI, USA). Right ventricular volumes were indexed to the body surface area (RVEDVI and RVESVI).

### Statistical analysis

All parametrically distributed data are expressed as the mean ± standard deviation (SD). Differences in blood flow parameters and RV function between patients with PAH and healthy volunteers were examined using the unpaired *t* test. Categorical variables were compared using Fisher’s exact test. Correlations between the two methods were assessed by Pearson’s correlation coefficient (r). The linear regression analysis was used to assess the correlation of VFT and WSS with PA dimensions, blood flow parameters and right ventricular functional variables. A *p* value <0.05 was considered statistically significant. All analyses were performed using SPSS statistics software (version 21.0; IBM Corporation, New York, NY, USA).

## Results

### Clinical characteristics

The nine PAH patients were consisted of five with idiopathic PAH, three with systemic lupus erythematosus-associated PAH, and one with congenital heart disease-associated PAH. Table 1 summarizes the subjects’ demographic and clinical characteristics. There were no significant differences in terms of age, sex, height, body weight or body surface area between patients with PAH and healthy volunteers. In patients with PAH, eight of nine patients were treated with a combination of a PDE-5 inhibitor and an ERA. One patient was not received any vasorelaxant agents. One PAH patient was excluded from the RV function analysis, because of missing data.

**Table 1:** Baseline characteristics of study participants

	Healthy volunteers	PAH patients	p value
Number, n	6	9	
Age, years	45.8 ± 8.9	41.8 ± 14.7	0.558
Gender, n (male/female)	4/2	1/8	0.089
Height, cm	163.2 ± 10.7	157.7 ± 5.8	0.288
Body weight, kg	60.4 ± 11.1	53.2 ± 6.4	0.234
Body mass index, kg/m^2^	22.0 ± 2.5	21.4 ± 2.0	0.635
Body surface area, m^2^	1.63 ± 0.20	1.52 ± 0.11	0.209
IPAH/APAH, n	N/A	5/4	
PDE-5 inhibitors, n (%)	N/A	8 (88.9)	
Endothelin receptor antagonists, n (%)	N/A	8 (88.9)	
Prostanoids, n (%)	N/A	5 (55.6)	

Data are expressed as n (%) or mean ± standard deviation

*PAH* pulmonary arterial hypertension, *IPAH* idiopathic pulmonary arterial hypertension, *APAH* associated pulmonary arterial hypertension, *PDE-5 inhibitors* phosphodiesterase 5 inhibitors, *N/A* not available

### Flow pattern

**Fig. 2:**
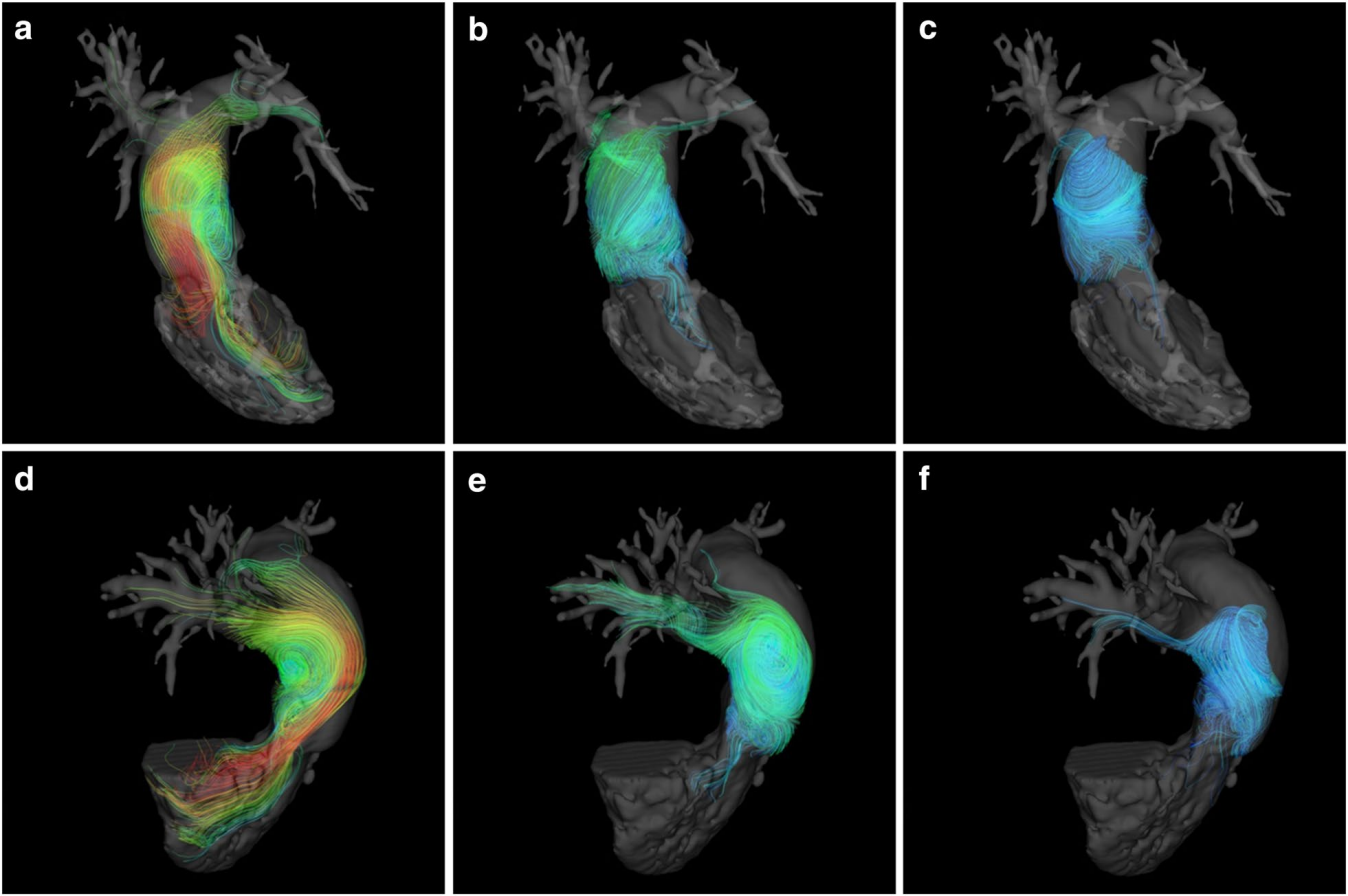
The blood flow in a patient with pulmonary arterial hypertension. Three-dimensional streamline visualizations of blood flow at peak systolic (**a**, **d**), mid diastolic (**b**, **e**) and end diastolic phases (**c**, **f**) in a patient with pulmonary arterial hypertension (**a**–**c**; in the right anterior oblique view, and **d**, **e**; in the left anterior oblique view). *Blue color* indicates 0 mm/s, and *red color* indicates maximum blood velocity. *LAO* left anterior oblique view, *RAO* right anterior oblique view, *MPA* main pulmonary artery, *LPA* left pulmonary artery, *RPA* right pulmonary artery

The 3D streamline images revealed the existence of vortex blood flow in the MPA in all patients with PAH. There was no inter-observer disagreement on the determination of vortex blood flow patterns. Typical images of 3D streamlines of the vortex appearance in PAH are shown in Fig. 2. The mean VFT was 35.0 ± 16.6 % of the cardiac cycle in PAH. The early systolic retrograde flow at the MPA was also seen in all patients with PAH, but this blood flow pattern was not seen in any of the healthy volunteers.

### Pulmonary artery dimensions and blood flow parameters

The arterial dimensions and the blood flow parameters obtained by 4D-flow at the MPA, RPA and LPA are shown in Table 2. Both MPA and RPA diameters, and RPA area in each cross-sectional plane were significantly greater in patients with PAH than healthy volunteers, but neither LPA diameter nor MPA and LPA areas differed significantly between the groups. Maximum blood flow velocities in the MPA, RPA and LPA in patients with PAH were significantly lower than healthy volunteers. Although mean blood flow velocity in the LPA in patients with PAH was not significantly different to that of healthy volunteers, mean blood flow velocities in the MPA and RPA were significantly lower. Maximum and mean blood flow volumes and SVs in the MPA, RPA and LPA did not differ significantly between the groups.

**Table 2:** Pulmonary artery dimensions and blood flow parameters in patients with PAH and healthy volunteers

	Healthy volunteers	PAH patients	p value
Diameter (mm)			
MPA	13.0 ± 1.0	15.6 ± 2.5	0.038
RPA	8.0 ± 0.7	11.7 ± 1.7	0.001
LPA	9.1 ± 0.8	13.8 ± 7.6	0.097
Area (mm^2^)			
MPA	537.6 ± 94.2	780.0 ± 272.2	0.058
RPA	233.7 ± 36.3	435.8 ± 132.2	0.003
LPA	262.3 ± 47.0	753.3 ± 897.8	0.209
Maximum blood flow velocity (m/sec)			
MPA	0.41 ± 0.10	0.28 ± 0.09	0.019
RPA	0.41 ± 0.15	0.22 ± 0.07	0.006
LPA	0.32 ± 0.10	0.18 ± 0.07	0.010
Mean blood flow velocity (m/sec)			
MPA	0.10 ± 0.02	0.07 ± 0.02	0.011
RPA	0.11 ± 0.04	0.06 ± 0.02	0.007
LPA	0.09 ± 0.02	0.06 ± 0.05	0.249
Maximum blood flow volume (L/min)			
MPA	13.54 ± 2.83	12.74 ± 3.49	0.650
RPA	5.87 ± 1.08	5.96 ± 1.05	0.874
LPA	5.10 ± 1.50	4.85 ± 2.83	0.911
Mean blood flow volume (L/min)			
MPA	3.14 ± 0.77	2.78 ± 0.34	0.240
RPA	1.53 ± 0.29	1.65 ± 0.55	0.618
LPA	1.38 ± 0.38	1.41 ± 0.62	0.909
Stroke volume (mL)			
MPA	62.5 ± 15.6	54.9 ± 7.3	0.219
RPA	30.5 ± 5.8	32.7 ± 10.6	0.667
LPA	27.4 ± 7.5	27.7 ± 12.0	0.945

Data are expressed as mean ± standard deviation

*PAH* pulmonary arterial hypertension, *MPA* main pulmonary artery, *RPA* right pulmonary artery, *LPA* left pulmonary artery

### Wall shear stress

**Fig. 3:**
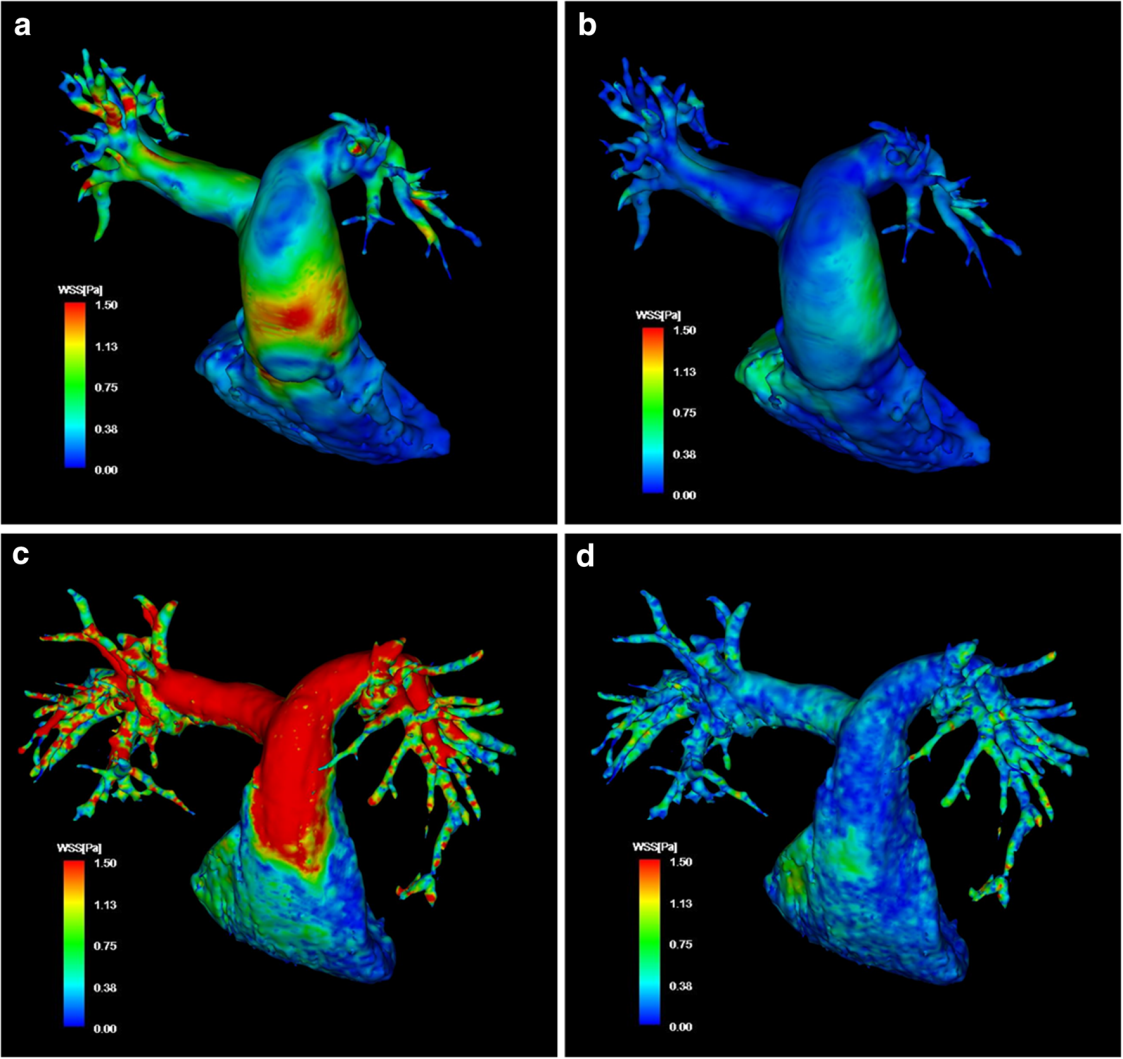
The wall shear stress visualization in a patient with pulmonary arterial hypertension. **a**, **b** Typical three-dimensional visualization of wall shear stress (WSS) at different cardiac phases in a patient with pulmonary arterial hypertension at peak systolic (**a**) and end diastolic phases (**b**). **c**, **d** For comparison, 3D visualizations of WSS in a healthy volunteer are shown at peak systolic (**c**) and end diastolic phases (**d**). Pulmonary arterial wall color indicates WSS; the color shift from *blue* to *red* denotes increases in WSS. *Blue color* indicates 0 Pa; *red color*, >1.5 Pa. *WSS* wall shear stress, *MPA* main pulmonary artery, *LPA* left pulmonary artery, *RPA* right pulmonary artery

Figure 3 shows 3D visualizations of WSS during the peak systolic and end diastolic phases in a patient with PAH and a healthy volunteer. The WSS in patients with PAH was spatially and temporally heterogeneous as compared with healthy volunteers. Maximum WSS in the pulmonary trunk, proximal RPA and proximal LPA, and mean WSS in the proximal RPA and proximal LPA, were significantly lower in patients with PAH than healthy volunteers; however, mean WSS in the pulmonary trunk did not differ significantly between the groups (Table 3).

**Table 3:** Wall shear stress in patients with PAH and healthy volunteers

	Healthy volunteers	PAH patients	p value
Maximum WSS (Pa)			
MPA	1.43 ± 0.35	0.93 ± 0.23	0.005
RPA	1.65 ± 0.68	0.77 ± 0.23	0.003
LPA	1.58 ± 0.50	0.64 ± 0.22	<0.001
Mean WSS (Pa)			
MPA	0.47 ± 0.07	0.46 ± 0.09	0.854
RPA	0.59 ± 0.13	0.35 ± 0.17	0.002
LPA	0.56 ± 0.08	0.39 ± 0.07	0.014

Data are expressed as mean ± standard deviation

*PAH* pulmonary arterial hypertension, *WSS* wall shear stress, *MPA* main pulmonary artery, *RPA* right pulmonary artery, *LPA* left pulmonary artery

### Right ventricular function

Table 4 shows the RV functional parameters obtained by 2D FIESTA cine images: RVEDVI was significantly greater in patients with PAH than healthy volunteers. Although RVEDV, RVESV and RVESVI tended to be higher in PAH, the differences were not statistically significant. Right ventricular ejection fraction was broadly comparable between the groups, and mean values in both groups were within normal range (40–60 %) of our calculation software.

**Table 4:** Right ventricular function in patients with PAH and healthy volunteers

	Healthy volunteers	PAH patients	p value
RVEF (%)	45.8 ± 6.7	43.5 ± 16.0	0.769
RVEDV (mL)	96.6 ± 26.8	135.3 ± 42.8	0.099
RVEDVI (mL/m^2^)	56.4 ± 13.7	90.7 ± 26.9	0.024
RVESV (mL)	55.5 ± 15.4	79.4 ± 41.1	0.244
RVESVI (mL/m^2^)	31.6 ± 5.24	53.0 ± 26.0	0.102

Data are expressed as mean ± standard deviation

*PAH* pulmonary arterial hypertension, *RVEF* right ventricular ejection fraction, *RVEDV* right ventricular end diastolic volume, *RVEDVI* right ventricular end diastolic volume index, *RVESV* right ventricular end systolic volume, *RVESVI* right ventricular end systolic volume index

### Relationships between duration of vortex and PA dimensions, blood flow parameters and right ventricular function

**Fig. 4:**
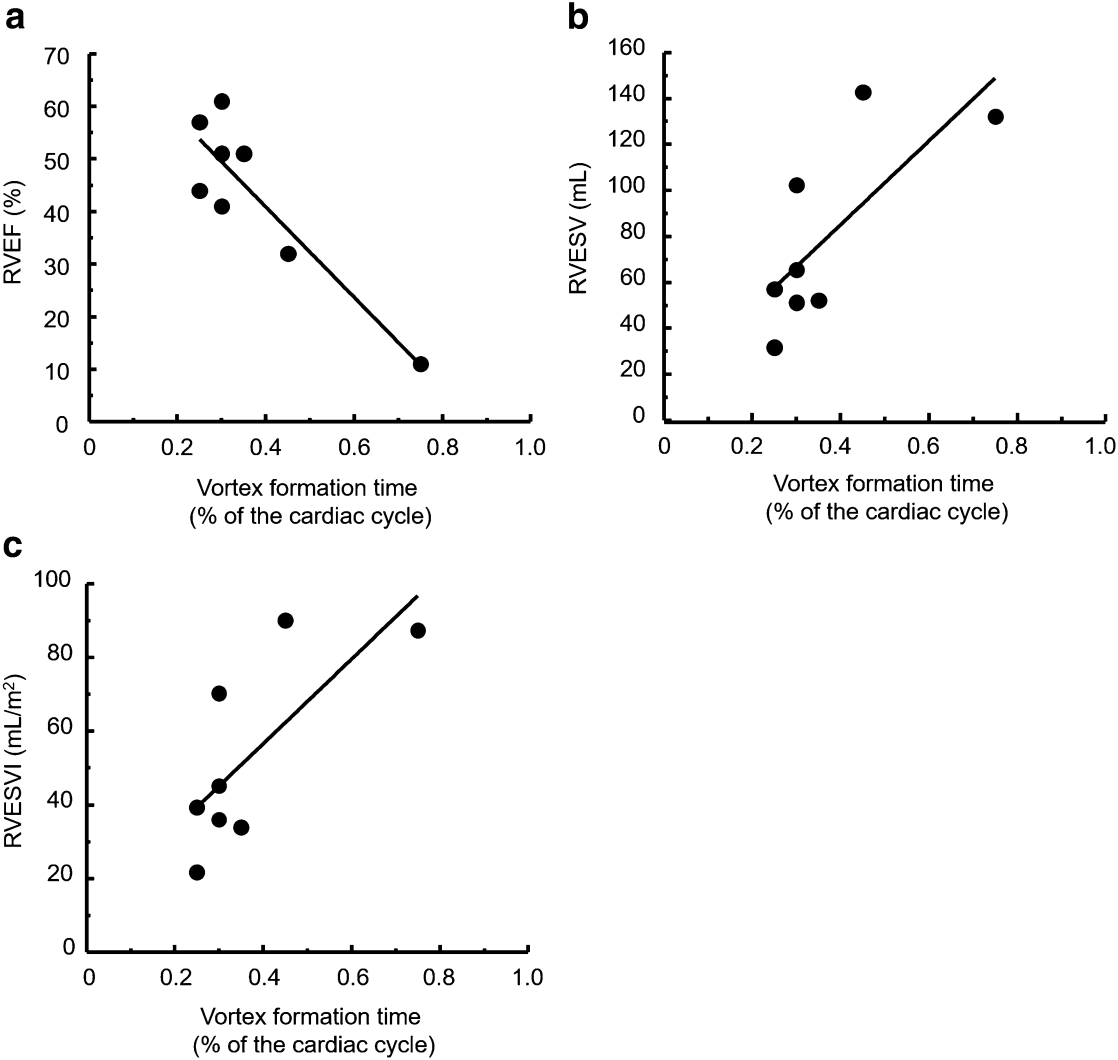
Scatter plot diagrams and regression analysis for correlation of right ventricular systolic function and vortex formation time in PAH patients. **a** Correlation between the right ventricular ejection fraction and the vortex formation time (model equation, RVEF = 75.1 + (−85.7)·VFT, p = 0.003). **b** Correlation between the right ventricular end systolic volume and the vortex formation time (model equation, RVESV = 12.4 + 181.8·VFT, p = 0.037). **c** Correlation between the right ventricular end systolic volume index and the vortex formation time (model equation, RVESVI = 10.6 + 114.8·VFT, p = 0.038)

The VFT, which is closely related to the mean PAP (Reiter et al. 2008, 2013), showed significantly negative correlation with RVEF, and significantly positive correlation with RVESV and RVESVI, whereas it did not correlate with PA dimensions, blood flow parameters or WSS in the MPA in PAH patients (Table 5). The corresponding linear equations were RVEF = 75.1 + (−85.7)·VFT, RVESV = 12.4 + 181.8·VFT and RVESVI = 10.6 + 114.8·VFT, respectively (Fig. 4). We also evaluated that whether both mean and maximum WSS in the MPA was associated with PA dimensions and RV function, whereas they did not correlate with either dimensions or RV function.

**Table 5:** Correlations of vortex formation time and wall shear stress with pulmonary artery dimensions, flow parameters and right ventricular function in patients with PAH

Variables		Correlation with vortex formation time (%cardiac cycle)		Correlation with maximum WSS at MPA (Pa)		Correlation with mean WSS at MPA (Pa)	
		r	p value	r	p value	r	p value
Geometry of MPA	Diameter (mm)	−0.257	0.504	0.035	0.928	−0.019	0.962
	Area (mm^2^)	−0.212	0.643	0.035	0.928	0.001	0.999
Blood flow parameter at MPA	Maximum blood flow velocity (m/sec)	−0.391	0.299	0.348	0.359	0.068	0.862
	Mean blood flow velocity (m/sec)	0.374	0.321	−0.091	0.817	−0.021	0.957
	Maximum blood flow volume (L/min)	−0.621	0.080	0.238	0.537	−0.115	0.768
	Mean blood flow volume (L/min)	−0.004	0.992	−0.119	0.760	−0.232	0.546
	SV (mL)	−0.025	0.948	−0.122	0.755	−0.262	0.496
WSS at MPA	Maximum WSS (pa)	−0.354	0.350	–	–	0.741	0.022
	Mean WSS (Pa)	−0.339	0.337	0.741	0.022	–	–
RV functional parameter	RVEF (%)	−0.892	0.003	0.546	0.162	0.592	0.112
	RVEDV (mL)	0.408	0.316	0.112	0.791	−0.417	0.305
	RVEDVI (mL/m^2^)	0.366	0.373	0.116	0.784	−0.427	0.291
	RVESV (mL)	0.738	0.037	−0.204	0.628	−0.525	0.182
	RVESVI (mL/m^2^)	0.736	0.038	−0.221	0.599	−0.546	0.162

*RV* right ventricle, *MPA* main pulmonary artery, *SV* stroke volume, *WSS* wall shear stress, *RVEF* right ventricular ejection fraction, *RVEDV* right ventricular end diastolic volume, *RVEDVI* right ventricular end diastolic volume index, *RVESV* right ventricular end systolic volume, *RVESVI* right ventricular end systolic volume index

## Discussion

We evaluated the blood flow patterns and characteristics (i.e., flow velocity, flow volume, WSS and SV) obtained by 4D-flow, and RV functional parameters obtained by 2D FIESTA cine imaging, in patients with PAH. We found: (1) both vortex blood flow and early systolic retrograde flow in the MPA in all patients with PAH; (2) lower PA flow velocities and WSS in patients with PAH than healthy volunteers, but that blood flow volumes in the MPA, RPA and LPA and SV in the MPA were broadly comparable between the groups; (3) RVEDVI was significantly greater in patients with PAH than healthy volunteers; (4) VFT significantly correlated with RVEF, RVESV and RVESVI, but not with PA size, other blood flow parameters or WSS.

In patients with PAH, CMR allows non-invasive assessment of right ventricular function, which has important prognostic value (Yamada et al. 2012). 4D-flow can evaluate arterial hemodynamics, such as blood flow velocity, flow volume and WSS in vivo (Reiter et al. 2008; Mano et al. 2013; Barker et al. 2014). Furthermore, this method is capable of clearly visualizing blood flow patterns (e.g. vortex formation) and WSS (Odagiri et al. 2014). Therefore, 4D-flow is a useful method of characterizing blood flow both qualitatively and quantitatively in vivo.

It was recognized that the vortex blood flow and early systolic retrograde flow were characteristics of PAH, and vortex blood flow seemed to be linked to systolic retrograde flow (Reiter et al. 2008; Helderman et al. 2011). In current study, we also observed both vortex blood flow and early systolic retrograde flow in the MPA in all patients with PAH, whereas these were not seen in healthy volunteers. Our results confirm the findings of previous reports.

Several studies have previously evaluated the blood flow parameters in PAH. In these studies, although the blood flow velocity measured in the PA was lower in PAH than normal subjects, it is still not clear whether there are differences in blood flow volume (Garcia-Alvarez et al. 2011; Helderman et al. 2011; Truong et al. 2013; Barker et al. 2014). In our study, blood flow velocity was reduced in patients with PAH compared with healthy volunteers; however, blood flow volume and SV did not differ between the groups. In our study PAH patients, their duration of disease was widespread with a range of 1–15 years, and they received various PAH treatments. These different stages and severities of disease and treatments could confound the study results. Indeed, standard deviation of LPA diameter was enormously high in our result, it was because one patients showed the large diameter LPA (area; 2966.2 mm^2^ and diameter; 30.7 mm, respectively), and her disease duration was 15 years. From our results, we speculated that dilatation of pulmonary artery might maintain blood flow volumes, even in the condition of decreased blood flow velocities. Boerrigter and colleagues reported that MPA dilatation was related to neither PAP nor cardiac output (Boerrigter et al. 2010). In contrast, it has been reported that in animal models an increased PAP was associated with structural changes in pulmonary atrial wall tissue, leading to remodeling of the PA (Kobs and Chesler 2006; Lammers et al. 2008). These findings supported that remodeling and dilatation of the proximal PA might represent a physiologic response that maintained pulmonary hemodynamics in PAH.

There are currently several methods of evaluating the severity of PAH in clinical practice. Transthoracic UCG is non-invasive and provides a useful estimation of PAP and tricuspid annular plane systolic excursion. Right heart cardiac catheterization is essential for the diagnosis of pulmonary hypertension. However, neither of these modalities can measure WSS. 4D-flow is a unique means of characterizing WSS qualitatively and quantitatively in PAH.

It is widely recognized that WSS influences endothelial cell function. Increasing WSS reportedly enhances endothelial nitric oxide synthase activity followed by vascular dilatation (Noris et al. 1995; Corson et al. 1996; Fisher et al. 2001). Although PAH is characterized by increased pulmonary vascular resistance (PVR) from remodeling and occlusion of the small pulmonary arterioles, previous reports indicated that systemic endothelial dysfunction observed in PAH (Wolff et al. 2007; Peled et al. 2008; Gabrielli et al. 2011). The endothelial-dependent flow-mediated vasodilation of the brachial artery was impaired in PAH patients (Wolff et al. 2007; Gabrielli et al. 2011), which indicated decrease of systemic nitric oxide bioavailability in PAH patients. In our cohort, WSS was lower in patients with PAH than healthy volunteers, a finding that is consistent with other investigators (Tang et al. 2012; Truong et al. 2013; Barker et al. 2014). However, the absolute values of WSS in both PAH patients and volunteers differed from previous papers (Barker et al. 2014; Tang et al. 2012). Both vessel diameters and flow velocities are determination factor of WSS values. The differences of WSS values were associated with heterogeneity of study populations, especially in PAH patients. They might have different disease stages, severities and receive various treatments. These clinical backgrounds might affect the estimated WSS values. In addition, pulmonary arterial wall segmentation, calculation algorithms also could affect WSS values. Wall shear stress is thought to reflect disease progression in PAH (Tang et al. 2012; Truong et al. 2013), and therefore may have potential as a biomarker for PAH, although further studies will be needed to confirm this hypothesis.

Analysis of the National Institutes of Health registry showed that increased mean PAP is associated with poor prognosis in PAH (D’Alonzo et al. 1991). Right heart failure is known to be a major complication of PAH, and it is well established that RV dysfunction also strongly influences mortality in PAH (D’Alonzo et al. 1991; Ghio et al. 2010). Other investigators have reported a correlation between VFT and mean PAP, and that the existence of a vortex in the PA combined with the VFT is diagnostic of PAH (Reiter et al. 2008, 2013, 2015). Nevertheless, the relationship between VFT and PA blood flow parameters is still not clear. Consequently, we explored whether VFT correlated with measures of RV function obtained by 2D FIESTA cine images and blood flow parameters obtained by 4D-flow in PAH. Our findings indicate that VFT is correlated with RV systolic dysfunction, but that other blood flow parameters, such as the flow volume, velocity and WSS, were not. Vortex formation time may therefore be a predictor of prognosis in PAH.

Our study has some important limitations. First, most of our patients were treated with PDE-5 inhibitors and ERAs, which dilate the PA and thus would be expected to influence blood flow parameters in PAH. Second, we did not evaluate the relationship between RHC and UCG, and 4D-flow data, because our subjects did not undergo RHC or UCG just before or after the 4D-flow imaging studies. Third, we did not measure blood viscosity. Fourth, RV volume parameters were automatically calculated in current study. It was reported that RV volumes were significantly affected by inclusion or exclusion of trabeculaions and papillary muscles (Kawel-Boehm et al. 2015). The operator drew a cavity of the RV in case of failure of the algorithm, however, the algorithm could affect the RV functional values. Fifth, PA pressure could also affect RV function, however, we could not confirm the correlation of VFT and PAP or PVR, because we did not evaluate the RHC data. Sixth, as described above, pharmaceutical treatments, disease stages, severities could be confounding factors of measurements, especially in PAH patients. Seventh, validation of 4D-flow was not fully established. Many studies have compared the technique to standard Doppler ultrasound (US) in various vessels. The quantification results of these studies generally showed lower velocities with 4D flow MRI compared to Doppler US (Harloff et al. 2009; Stankovic et al. 2012) with a moderate correlation between the methods. It is possible that this could affect the interpretation of our results. Finally, our sample size was small, which affects the power of statistical tests and increases the risk that a type II error could have occurred.

## Conclusion

Pulmonary blood flow velocity and WSS in patients with PAH were lower than healthy volunteers, and these findings were consistent with previous reports. We also confirmed that abnormal blood flow dynamics, including vortex formation and early onset of retrograde flow were characteristic of PAH. We found that the VFT was associated with RV systolic function in PAH patients. Further prospective studies are necessary to identify and validate whether WSS and VTF are clinically useful biomarkers for PAH.

## Additional files

10.1186/s40064-016-2755-7 The supplemental method: MRI protocol and imaging parameters.

## Abbreviations

PAH: pulmonary arterial hypertension
PAP: pulmonary arterial pressure
PDE-5: phosphodiesterase-5
ERA: endothelin receptor antagonists
UCG: echocardiography
CMR: cardiac magnetic resonance imaging
RV: right ventricle
3D: 3-dimension
WSS: wall shear stress
VFT: vortex formation time
RHC: right heart cardiac catheterization
PA: pulmonary artery
MPA: main pulmonary artery
RPA: right pulmonary artery
LPA: left pulmonary artery
WHO: World Health Organization
MRA: magnetic resonance angiography
FIESTA: fast cine steady state free precession
SV: stroke volume
RVEDV: right ventricular end diastolic volume
RVESV: right ventricular end systolic volume
RVEF: right ventricular ejection fraction
RVEDVI: right ventricular end diastolic volume index
RVESVI: right ventricular end systolic volume index
SD: standard deviation
PVR: pulmonary vascular resistance

## References

[CR1] Barker AJ, Roldan-Alzate A, Entezari P, Shah SJ, Chesler NC, Wieben O, Markl M, Francois CJ (2014). Four-dimensional flow assessment of pulmonary artery flow and wall shear stress in adult pulmonary arterial hypertension: results from two institutions. Magn Reson Med.

[CR2] Boerrigter B, Mauritz GJ, Marcus JT, Helderman F, Postmus PE, Westerhof N, Vonk-Noordegraaf A (2010). Progressive dilatation of the main pulmonary artery is a characteristic of pulmonary arterial hypertension and is not related to changes in pressure. Chest.

[CR3] Bradlow WM, Gibbs JS, Mohiaddin RH (2012). Cardiovascular magnetic resonance in pulmonary hypertension. J Cardiovasc Magn Reson.

[CR4] Cheng CP, Parker D, Taylor CA (2002). Quantification of wall shear stress in large blood vessels using Lagrangian interpolation functions with cine phase-contrast magnetic resonance imaging. Ann Biomed Eng.

[CR5] Corson MA, James NL, Latta SE, Nerem RM, Berk BC, Harrison DG (1996). Phosphorylation of endothelial nitric oxide synthase in response to fluid shear stress. Circ Res.

[CR6] D’Alonzo GE, Barst RJ, Ayres SM, Bergofsky EH, Brundage BH, Detre KM, Fishman AP, Goldring RM, Groves BM, Kernis JT (1991). Survival in patients with primary pulmonary hypertension. Results from a national prospective registry. Ann Intern Med.

[CR7] Fisher AB, Chien S, Barakat AI, Nerem RM (2001). Endothelial cellular response to altered shear stress. Am J Physiol Lung Cell Mol Physiol.

[CR8] Fisher MR, Forfia PR, Chamera E, Housten-Harris T, Champion HC, Girgis RE, Corretti MC, Hassoun PM (2009). Accuracy of Doppler echocardiography in the hemodynamic assessment of pulmonary hypertension. Am J Respir Crit Care Med.

[CR9] Gabrielli LA, Castro PF, Godoy I, Mellado R, Bourge RC, Alcaino H, Chiong M, Greig D, Verdejo HE, Navarro M, Lopez R, Toro B, Quiroga C, Diaz-Araya G, Lavandero S, Garcia L (2011). Systemic oxidative stress and endothelial dysfunction is associated with an attenuated acute vascular response to inhaled prostanoid in pulmonary artery hypertension patients. J Card Fail.

[CR10] Galie N, Ghofrani HA, Torbicki A, Barst RJ, Rubin LJ, Badesch D, Fleming T, Parpia T, Burgess G, Branzi A, Grimminger F, Kurzyna M, Simonneau G, Sildenafil Use in Pulmonary Arterial Hypertension Study G (2005). Sildenafil citrate therapy for pulmonary arterial hypertension. N Engl J Med.

[CR11] Galie N, Hoeper MM, Humbert M, Torbicki A, Vachiery JL, Barbera JA, Beghetti M, Corris P, Gaine S, Gibbs JS, Gomez-Sanchez MA, Jondeau G, Klepetko W, Opitz C, Peacock A, Rubin L, Zellweger M, Simonneau G (2009). Guidelines for the diagnosis and treatment of pulmonary hypertension. Eur Respir J.

[CR12] Garcia-Alvarez A, Fernandez-Friera L, Mirelis JG, Sawit S, Nair A, Kallman J, Fuster V, Sanz J (2011). Non-invasive estimation of pulmonary vascular resistance with cardiac magnetic resonance. Eur Heart J.

[CR13] Ghio S, Klersy C, Magrini G, D’Armini AM, Scelsi L, Raineri C, Pasotti M, Serio A, Campana C, Vigano M (2010). Prognostic relevance of the echocardiographic assessment of right ventricular function in patients with idiopathic pulmonary arterial hypertension. Int J Cardiol.

[CR14] Harloff A, Albrecht F, Spreer J, Stalder AF, Bock J, Frydrychowicz A, Schollhorn J, Hetzel A, Schumacher M, Hennig J, Markl M (2009). 3D blood flow characteristics in the carotid artery bifurcation assessed by flow-sensitive 4D MRI at 3T. Magn Reson Med.

[CR15] Helderman F, Mauritz GJ, Andringa KE, Vonk-Noordegraaf A, Marcus JT (2011). Early onset of retrograde flow in the main pulmonary artery is a characteristic of pulmonary arterial hypertension. J Magn Reson Imaging.

[CR16] Isoda H, Ohkura Y, Kosugi T, Hirano M, Alley MT, Bammer R, Pelc NJ, Namba H, Sakahara H (2010). Comparison of hemodynamics of intracranial aneurysms between MR fluid dynamics using 3D cine phase-contrast MRI and MR-based computational fluid dynamics. Neuroradiology.

[CR17] Kawel-Boehm N, Maceira A, Valsangiacomo-Buechel ER, Vogel-Claussen J, Turkbey EB, Williams R, Plein S, Tee M, Eng J, Bluemke DA (2015). Normal values for cardiovascular magnetic resonance in adults and children. J Cardiovasc Magn Reson.

[CR18] Kobs RW, Chesler NC (2006). The mechanobiology of pulmonary vascular remodeling in the congenital absence of eNOS. Biomech Model Mechanobiol.

[CR19] Lammers SR, Kao PH, Qi HJ, Hunter K, Lanning C, Albietz J, Hofmeister S, Mecham R, Stenmark KR, Shandas R (2008). Changes in the structure-function relationship of elastin and its impact on the proximal pulmonary arterial mechanics of hypertensive calves. Am J Physiol Heart Circ Physiol.

[CR20] Liu Z, Moorhead RJ, Groner J (2006). An advanced evenly-spaced streamline placement algorithm. IEEE Trans Visual Comput Graph.

[CR21] Mano Y, Takehara Y, Sakaguchi T, Alley MT, Isoda H, Shimizu T, Wakayama T, Sugiyama M, Sakahara H, Konno H, Unno N (2013). Hemodynamic assessment of celiaco-mesenteric anastomosis in patients with pancreaticoduodenal artery aneurysm concomitant with celiac artery occlusion using flow-sensitive four-dimensional magnetic resonance imaging. Eur J Vasc Endovasc Surg.

[CR22] Masaryk AM, Frayne R, Unal O, Krupinski E, Strother CM (1999). In vitro and in vivo comparison of three MR measurement methods for calculating vascular shear stress in the internal carotid artery. AJNR Am J Neuroradiol.

[CR23] McGoon M, Gutterman D, Steen V, Barst R, McCrory DC, Fortin TA, Loyd JE, American College of Chest P (2004). Screening, early detection, and diagnosis of pulmonary arterial hypertension: ACCP evidence-based clinical practice guidelines. Chest.

[CR24] McLaughlin VV, Archer SL, Badesch DB, Barst RJ, Farber HW, Lindner JR, Mathier MA, McGoon MD, Park MH, Rosenson RS, Rubin LJ, Tapson VF, Varga J, Harrington RA, Anderson JL, Bates ER, Bridges CR, Eisenberg MJ, Ferrari VA, Grines CL, Hlatky MA, Jacobs AK, Kaul S, Lichtenberg RC, Lindner JR, Moliterno DJ, Mukherjee D, Pohost GM, Rosenson RS, Schofield RS, Shubrooks SJ, Stein JH, Tracy CM, Weitz HH, Wesley DJ, Accf Aha (2009). ACCF/AHA 2009 expert consensus document on pulmonary hypertension: a report of the American College of Cardiology Foundation Task Force on Expert Consensus Documents and the American Heart Association: developed in collaboration with the American College of Chest Physicians, American Thoracic Society, Inc., and the Pulmonary Hypertension Association. Circulation.

[CR25] Noris M, Morigi M, Donadelli R, Aiello S, Foppolo M, Todeschini M, Orisio S, Remuzzi G, Remuzzi A (1995). Nitric oxide synthesis by cultured endothelial cells is modulated by flow conditions. Circ Res.

[CR26] Odagiri K, Inui N, Miyakawa S, Hakamata A, Wei J, Takehara Y, Sakahara H, Sugiyama M, Alley MT, Tran QK, Watanabe H (2014). Abnormal hemodynamics in the pulmonary artery seen on time-resolved 3-dimensional phase-contrast magnetic resonance imaging (4D-flow) in a young patient with idiopathic pulmonary arterial hypertension. Circ J.

[CR27] Ota H, Sugimura K, Miura M, Shimokawa H (2015). Four-dimensional flow magnetic resonance imaging visualizes drastic change in vortex flow in the main pulmonary artery after percutaneous transluminal pulmonary angioplasty in a patient with chronic thromboembolic pulmonary hypertension. Eur Heart J.

[CR28] Peled N, Bendayan D, Shitrit D, Fox B, Yehoshua L, Kramer MR (2008). Peripheral endothelial dysfunction in patients with pulmonary arterial hypertension. Respir Med.

[CR29] Pulido T, Adzerikho I, Channick RN, Delcroix M, Galie N, Ghofrani HA, Jansa P, Jing ZC, Le Brun FO, Mehta S, Mittelholzer CM, Perchenet L, Sastry BK, Sitbon O, Souza R, Torbicki A, Zeng X, Rubin LJ, Simonneau G, Investigators S (2013). Macitentan and morbidity and mortality in pulmonary arterial hypertension. N Engl J Med.

[CR30] Reiter G, Reiter U, Kovacs G, Kainz B, Schmidt K, Maier R, Olschewski H, Rienmueller R (2008). Magnetic resonance-derived 3-dimensional blood flow patterns in the main pulmonary artery as a marker of pulmonary hypertension and a measure of elevated mean pulmonary arterial pressure. Circ Cardiovasc Imaging.

[CR31] Reiter U, Reiter G, Kovacs G, Stalder AF, Gulsun MA, Greiser A, Olschewski H, Fuchsjager M (2013). Evaluation of elevated mean pulmonary arterial pressure based on magnetic resonance 4D velocity mapping: comparison of visualization techniques. PLoS One.

[CR32] Reiter G, Reiter U, Kovacs G, Olschewski H, Fuchsjager M (2015). Blood flow vortices along the main pulmonary artery measured with MR imaging for diagnosis of pulmonary hypertension. Radiology.

[CR33] Rubin LJ, Badesch DB, Barst RJ, Galie N, Black CM, Keogh A, Pulido T, Frost A, Roux S, Leconte I, Landzberg M, Simonneau G (2002). Bosentan therapy for pulmonary arterial hypertension. N Engl J Med.

[CR34] Rubin LJ, Badesch DB, Fleming TR, Galie N, Simonneau G, Ghofrani HA, Oakes M, Layton G, Serdarevic-Pehar M, McLaughlin VV, Barst RJ, Group S-S (2011). Long-term treatment with sildenafil citrate in pulmonary arterial hypertension: the SUPER-2 study. Chest.

[CR35] Runo JR, Loyd JE (2003). Primary pulmonary hypertension. Lancet.

[CR36] Stankovic Z, Csatari Z, Deibert P, Euringer W, Blanke P, Kreisel W, Abdullah Zadeh Z, Kallfass F, Langer M, Markl M (2012). Normal and altered three-dimensional portal venous hemodynamics in patients with liver cirrhosis. Radiology.

[CR37] Tang BT, Pickard SS, Chan FP, Tsao PS, Taylor CA, Feinstein JA (2012). Wall shear stress is decreased in the pulmonary arteries of patients with pulmonary arterial hypertension: an image-based, computational fluid dynamics study. Pulm Circ.

[CR38] Truong U, Fonseca B, Dunning J, Burgett S, Lanning C, Ivy DD, Shandas R, Hunter K, Barker AJ (2013). Wall shear stress measured by phase contrast cardiovascular magnetic resonance in children and adolescents with pulmonary arterial hypertension. J Cardiovasc Magn Reson.

[CR39] Wolff B, Lodziewski S, Bollmann T, Opitz CF, Ewert R (2007). Impaired peripheral endothelial function in severe idiopathic pulmonary hypertension correlates with the pulmonary vascular response to inhaled iloprost. Am Heart J.

[CR40] Yamada Y, Okuda S, Kataoka M, Tanimoto A, Tamura Y, Abe T, Okamura T, Fukuda K, Satoh T, Kuribayashi S (2012). Prognostic value of cardiac magnetic resonance imaging for idiopathic pulmonary arterial hypertension before initiating intravenous prostacyclin therapy. Circ J.

