# Supplementary material for: Non-invasive evaluation of pulmonary arterial blood flow and wall shear stress in pulmonary arterial hypertension with 3D phase contrast magnetic resonance imaging
Source: Springerplus. 2016 Jul 13;5(1):1071. doi: 10.1186/s40064-016-2755-7 (PMC4943915; doi:10.1186/s40064-016-2755-7)
Supplement: Supplementary file 1 — 10.1186/s40064-016-2755-7 The supplemental method: MRI protocol and imaging parameters. [file 40064_2016_2755_MOESM1_ESM.docx]

**Supplemental Data**

**Supplementary method**

**Time-resolved contrast-enhanced 3D MRA**

Contrast agents

Either Gd-DTPA-BMA (Omniscan, Daiichi Sankyo Company, Limited, Tokyo, Japan) or Gd-DTPA (Magnevist, Bayer Healthcare, Osaka, Japan) was used at a standard dose of 0.1 mmol/kg at an injection rate of 2–3 ml/s using an auto-injector (Sonic Shot GX, Nemoto Kyorindo, Tokyo, Japan), followed by a 20 ml flush of 0.9% NaCl solution at the same rate.

Imaging parameters for coronal 3D FSPGR MRA

Imaging parameters used were as follows: repetition time (TR; ms) / echo time (TE; ms) / flip angle (FA; degree) / number of excitations (NEX), 2.7 / 1.0 / 12 / 1; field of view (FOV), 32 cm; reconstructed matrix with the aid of zero filling interpolation, 224 × 224; receiver band width (RBW), 83.3 kHz; imaging time, 33 sec for four phases.

**Imaging parameters for 4D-Flow**

Electrocardiogram-gated, respiratory-compensated coronal 3D FSPGR-based 4D-Flow was used to image the right atrium, right ventricle and proximal portion of the PA. Imaging parameters used were as follows: TR (ms) / TE (ms) / FA (degree) / NEX, 4.5–5.0 / 2.0 / 15 / 1; FOV, 32 cm; matrix, 224 × 224, 2 mm thickness, 60 partitions, 20 phases during one cardiac cycle; velocity encoding (VENC), 200 cm/s; RBW, 83.3 kHz. Respiratory-compensated retrospective cardiac gating was also combined. The approximate imaging time was 10 min, with a reduction factor of two for an auto-calibrating reconstruction for Cartesian (ARC) sampling. Raw 4D-Flow data were transferred to a personal computer (Intel Xeon E3-1270 (3.4 GHz/Qad-core) DDR3, 16 GB ECC, Linux) and reconstructed.

**Calculation of WSS**

Shearing velocity was calculated by dividing velocity along the wall by distance from wall to the velocity measuring point. WSS is defined as the multiplication of fluid viscosity and shearing velocity of the neighboring vascular wall. We chose one WSS calculation point (S0) and three reference points (S1 to S3) at regular interval d of the normal to the wall. S0 was located at the wall and S1 to S3 were located in the vascular structure. Velocity vectors at S1, S2, and S3 were linearly interpolated by the surrounding data points with the use of linear interpolation method. The slope of the curve (derivative value) of the velocity vector profile at S0 was the velocity vector gradient at S0. We set the velocity at S0 to zero, and the velocity vector gradient at the wall was estimated with Lagrange’s polynomial interpolation formula. WSS vector at S0 was calculated by multiplying viscosity by the tangential velocity vector gradient perpendicular to the normal to the wall (i.e., tangential shearing velocity vector). WSS was the strength of the WSS vector.

**Imaging parameters for the 2D fast imaging employing steady state acquisition (FIESTA) cine images**

The imaging parameters were as follows: TR(ms)/TE(ms) was 3.1-3.4/1.3-1.5; matrix, 192 × 192; FOV, 34 cm; flip angle, 45°; 20 phases were reconstructed for each cardiac cycle; readout bandwidth, 125 kHz. The slice thickness/gap was typically 10 mm/0 mm (6-9 slices). Sixteen data lines were acquired for each segment.

**Supplementary reference**

**Figure Legends**

**Figure 1.** **Cross-sectional planes traversing the main, right and left pulmonary artery and the locations of the pulmonary trunk, proximal RPA and proximal LPA.**

(A) Cross-sectional planes traversing the main, right and left pulmonary artery. All cross-sectional planes were placed perpendicular to the longitudinal axis of the pulmonary artery. Each cut plane was placed immediately downstream of the pulmonary valve cusps or the bifurcation of the main pulmonary artery. (B and C)The locations of the pulmonary trunk (B), proximal RPA and proximal LPA (C), in which wall shear stress values were calculated.

MPA, main pulmonary artery; RPA, right pulmonary artery; LPA, left pulmonary artery, RPA, right pulmonary artery; LPA, left pulmonary artery

**Figure 2. The blood flow in a patient with pulmonary arterial hypertension**

Three-dimensional streamline visualizations of blood flow at peak systolic (A and D), mid diastolic (B and E) and end diastolic phases (C and F) in a patient with pulmonary arterial hypertension (A-C; in the right anterior oblique view, and D-E; in the left anterior oblique view). Blue color indicates 0 mm/s, and red color indicates maximum blood velocity.

LAO, left anterior oblique view; RAO, right anterior oblique view; MPA, main pulmonary artery; LPA, left pulmonary artery; RPA, right pulmonary artery.

**Figure 3. The wall shear stress visualization in a patient with pulmonary arterial hypertension**

(A and B) Typical three-dimensional visualization of wall shear stress (WSS) at different cardiac phases in a patient with pulmonary arterial hypertension at peak systolic (A) and end diastolic phases (B). (C and D) For comparison, 3D visualizations of WSS in a healthy volunteer are shown at peak systolic (C) and end diastolic phases (D). Pulmonary arterial wall color indicates WSS; the color shift from blue to red denotes increases in WSS. Blue color indicates 0 Pa; red color, >1.5 Pa.

WSS, wall shear stress; MPA, main pulmonary artery; LPA, left pulmonary artery; RPA, right pulmonary artery

**Figure 4. Scatter plot diagrams and regression analysis for correlation of right ventricular systolic function and vortex formation time in PAH patients.**

(A) Correlation between the right ventricular ejection fraction and the vortex formation time (model equation, RVEF=75.1+(-85.7)･VFT, p=0.003). (B) Correlation between the right ventricular end systolic volume and the vortex formation time (model equation, RVESV=12.4+181.8･VFT, p=0.037). (C) Correlation between the right ventricular end systolic volume index and the vortex formation time (model equation, RVESVI=10.6+114.8･VFT, p=0.038).
